# Study of kerosene caustic wash process for jet fuel production

**DOI:** 10.1186/s44147-021-00029-5

**Published:** 2021-11-11

**Authors:** Ahmed Mohamed Selim Abdelhamid, Hamdy Abdel-Aziz Mustafa

**Affiliations:** 1 Nasr Petroleum Company, Suez, Egypt; 2 Faculty of Engineering, Cairo University, Cairo, Egypt

**Keywords:** Kerosene, Jet fuel, Jet A-1, Total acidity, Caustic wash, Water content

## Abstract

Caustic wash is one of many industrial processes that are used to produce jet fuel. In this study, an analysis of the key parameters of the kerosene caustic wash process was conducted to improve the total performance of the treatment process. The investigated parameters are caustic concentration (from 0.03 to 3.0 wt%), caustic volume (from 110% of theoretical to 250%), number of treatment stages (one and two stages), wash water type (demineralized water and alkaline soft water), and wash water volume (10% and 30% of kerosene feed volume). Results revealed that the reaction between sodium hydroxide and naphthenic acids is a diffusion-controlled chemical reaction. The diluted caustic solutions (0.5 wt%) are better than the concentrated ones (3 wt%). Higher excess caustic volume has a slight effect on kerosene acidity. Performing the caustic treatment process in one stage is sufficient, and the two-stage process has no effect on acidity. Washing caustic-treated kerosene with demineralized water (pH=7) has a slight adverse effect on kerosene acidity. Increasing the demineralized water volume results in a slight increase in the acidity of the treated kerosene. Wash water should be slightly alkaline (pH 7.5–8) to prevent the reverse reaction of sodium naphthenates back into naphthenic acid. Increasing wash water volume (more than 10 vol% of kerosene feed) has no noticeable effect on the water content of treated kerosene.

## Introduction

The aviation sector is a fast-growing transportation sector, although it faces big challenges today due to COVID-19. The worldwide airline operations consume annually around 1500–1700 million barrels of Jet A-1 fuel. Forecasts indicate that the aviation sector will grow at 4.8% per year until 2036. Airlines from all over the world must purchase quality and safe fuels, and hence, jet fuel must meet very restricted international specifications [1, 2]. International Norms establish the quality specifications of the jet fuel: ASTM D-1655 and DEF STAN 91-91. Table 1 lists the standard specifications for kerosene-type aviation turbine fuel (Jet A-1) [3–5].

**Table 1:** Standard specifications for kerosene-type aviation turbine fuel (Jet A-1) [3–5]

Property	Limits
Visual appearance	Clear, bright, and visually free from solid matter and un-dissolved water at ambient fuel temperature
Total acidity, mg KOH/g	0.015 max.
Sulphur, total, % m/m	0.30 max.
Sulphur, mercaptan, % m/m	0.0030 max.
End point, °C	300 max.
Flash point, °C	38 min.
Density at 15°C, kg/m^3^	775.0 to 840.0
Freezing point, °C	−47 max.
Specific energy, net, MJ/kg	42.80 min.
Smoke point, mm	25 min.
Aromatics, % v/v.	25 max.

*Min.* minimum, *max.* maximum

Many industrial processes are used to produce jet fuels with those specifications, based on the impurities in the kerosene. Among these processes are the caustic wash process, UOP caustic-free Merox process, Merichem Napfining and MERICAT processes, hydrotreating, and alternative renewable jet fuels [6–12].

The caustic wash process is limited to refineries that produce kerosene fractions which already meet the international jet fuel specifications except for the total acidity. The process consists of withdrawing a side-stream kerosene from the atmospheric crude distillation unit followed by stripping, cooling, caustic washing, water washing, salt drying, clay filtration, and final water separation. Total acidity is the only specification that is caustic-extractable. Other specifications such as aromatics, smoke point, sulfur content, and freezing point are not caustic-extractable and hence not affected by the caustic wash process [2, 6].

Water content is an important parameter that reflects the fuel purity. Water content, in the dissolved phase alone, does not affect fuel performance. However, water in any other phase could participate in aircraft incidents and accidents. The excess water content affects directly the fuel quality and the normal operation of the flight equipment, even severely endangering the flight safety. Free water can affect the aircraft’s fuel system reliability and lead to operational delays and increased maintenance costs [13–17].

Many improvements have been done to enhance the caustic wash performance. The Fiber-Film Contactor employs non-dispersive contacting of the caustic and hydrocarbon phases. This prevents emulsion formation and minimizes caustic and water carryover. The contactor provides a large interfacial surface area which increases the mass transfer rate [2, 10]. The sodium hydroxide solution of ethanol was used as the acid removal reagent. This process was introduced to solve the problem of emulsion formation associated with aqueous sodium hydroxide [18].

In this study, an analysis of the key parameters of the kerosene caustic wash process was conducted to improve the total performance of the treatment process (minimizing caustic consumption, minimizing wash water consumption, and minimizing residual water carried-over in the treated product). The investigated parameters are caustic concentration, caustic volume, number of treatment stages, wash water type, and wash water volume. Focus is placed on reducing the total acidity of petroleum kerosene to meet Jet A-1 specifications.

## Methods

In this study, the main target is to analyze the caustic wash process of petroleum kerosene fractions to produce jet fuel matching the international standard specifications of Jet A-1 (ASTM D-1655 and DEF STAN 91-91) [4, 5].

### Materials

A sample of straight-run kerosene was taken from the atmospheric distillation unit. Table 2 summarizes the properties of this sample. Two types of wash water were used, demineralized water and alkaline soft water. Table 3 summarizes the properties of both types.

**Table 2:** Properties of kerosene sample

Total acidity mg KOH/g	0.044
Specific gravity 60/60 ^0^F	0.7933
Total sulfur, wt%	0.12
Water content, ppm	56
ASTM distillation D-86	
Initial boiling point, ^0^C	145
10% volume distilled at, ^0^C	159
30% volume distilled at, ^0^C	173
50% volume distilled at, ^0^C	188
70% volume distilled at, ^0^C	208
90% volume distilled at, ^0^C	233
Final boiling point, ^0^C	256

**Table 3:** Properties of wash water

Water type	Demineralized water	Alkaline soft water
pH	7	9.44
Total dissolved solids (ppm)	0	330
Total alkalinity (ppm as CaCO_3_)	-----	24
P alkalinity (ppm as CaCO_3_)	-----	0.6
M alkalinity (ppm as CaCO_3_)	-----	1.2
OH^-^ alkalinity (ppm as CaCO_3_)	-----	Nil
CO3^—^alkalinity (ppm as CaCO_3_)	-----	24
HCO_3_^-^ alkalinity (ppm as CaCO_3_)	-----	Nil

### Chemicals, reagents, tests, and analytical equipment

All chemicals used were of analytical higher grades. The total acidity test was carried out using the ASTM method D-3242 standard. The water content of kerosene was measured by Coulometric Karl Fischer Titration (Karl-Fischer Moisture Titrator MKC-520–KEM, Co.). The water content of the sample was measured by three parallel experiments, and the maximum value was reported [14]. Wash water pH and TDS were measured by Mettler-Toledo AG FiveEasy^TM^ Plus FEB30.

### Methodology of kerosene treatment

**Fig. 1:**
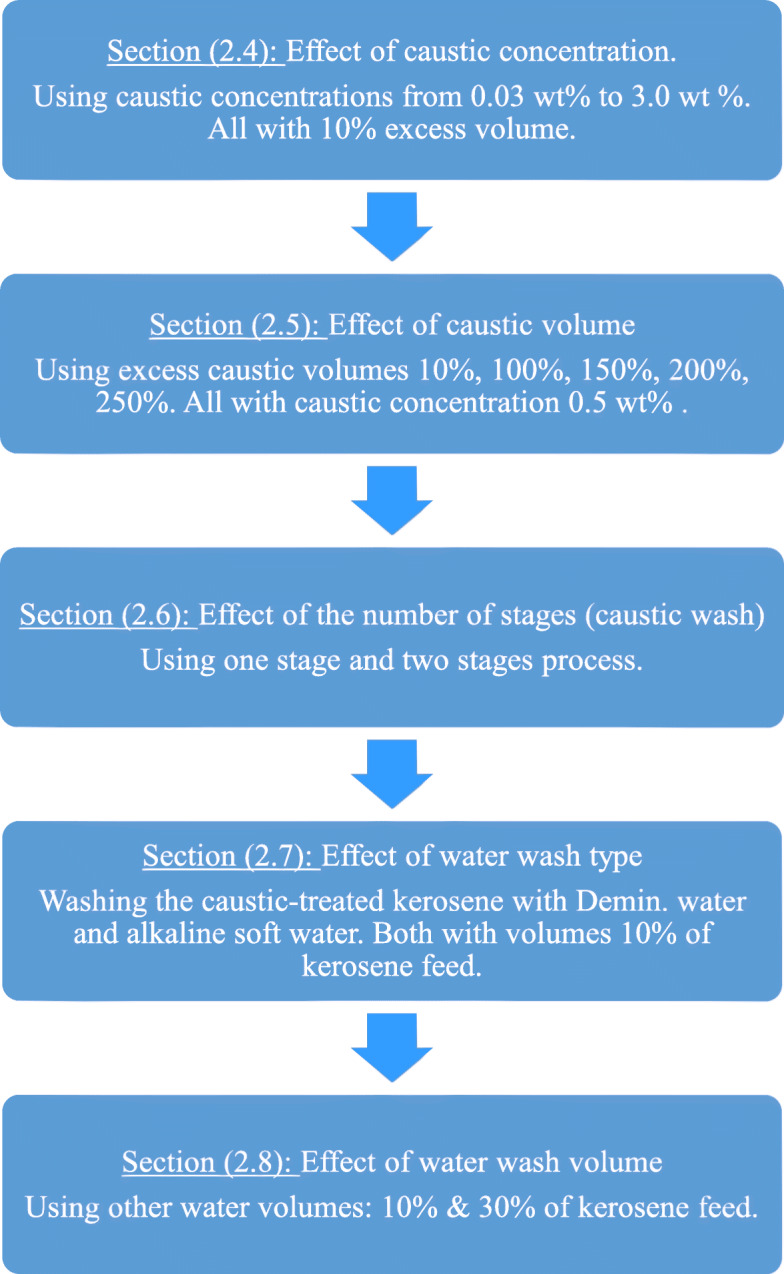
Flow chart of all steps in the current study

One liter of kerosene feed (Table 2) is mixed with a calculated volume of caustic solution and stirred together for 5 min with a 300 RPM laboratory mixer. The mixture is settled for 30 min for separation (by gravity) of the aqueous phase from the “treated” kerosene phase. Figure 1 illustrates a flow chart of the overall steps in the current study.

### Effect of caustic concentration on the treatment process

Different caustic concentrations were used: 0.03 wt%, 0.05 wt%, 0.125 wt%, 0.25 wt%, 0.5 wt%, 1 wt%, and 3 wt%. The volume of the caustic solution depends on the caustic concentration. Table 4 indicates the volume of caustic solutions used in the treatment process. As the caustic concentration increases, the stoichiometric caustic volume decreases. Excess caustic is added to ensure a complete reaction. In this stage, 10% excess caustic (110% of the theoretical) is used. The effect of excess caustic will be studied later in section 2.5. The treatment process as per section 2.3 was performed to measure the total acidity of treated kerosene.

**Table 4:** Volume of caustic solution used in the treatment process

Caustic concentration	Vol. of caustic solution (^a^)	Vol. of feed sample
0.03 wt %	92 ml	1 L
0.05 wt %	55 ml	1 L
0.125 wt %	22 ml	1 L
0.25 wt %	11 ml	1 L
0.5 wt %	5.5 ml	1 L
1.0 wt %	2.8 ml	1 L
3.0 wt %	1 ml	1 L

^a^Including 10% excess

### Effect of caustic volume on the treatment process

Kerosene sample was treated using the caustic solution with concentration 0.5 wt% and different excess volumes of caustic: 10%, 100%, 150%, 200%, and 250%. The total acidity of treated kerosene was then measured.

### Effect of the number of treatment stages on the treatment process.

Kerosene sample was treated with caustic solution 1 wt% in two stages (using 10% excess caustic) to study the effect of the number of treatment stages on the effectiveness of the treatment process. The total acidity of the treated kerosene was measured and compared with the results of the one-stage process.

### Effect of water wash on the treatment process

In the industrial plants, the caustic wash is normally followed by water wash to remove any entrained droplets of caustic solution that escapes with the treated kerosene and impairs the downstream systems. Here, caustic wash followed by water wash will be studied. Two types of wash water were used, demineralized water and alkaline soft water. Table 3 summarizes the properties of both types of wash water. Step (1) of caustic wash is carried out as per section 2.3 using the caustic solution with concentration 1 wt% and 10% excess caustic. Step (2) of water wash was carried out using the volume of water equal to 10% of kerosene feed (100 ml wash water per 1 l of kerosene feed).

Caustic-washed kerosene (from step 1) is washed with water by stirring together with a 300 RPM laboratory mixer for 5 min. The mixture is left for 30 min for separation of the aqueous phase (by gravity) from the “treated” kerosene. Total acidity and water content of treated kerosene were measured and recorded.

### Effect of wash water volume percent on the treatment process

The volume of wash water has increased from 10 to 30%. The treatment process as per section 2.7 was performed. Total acidity and water content of treated kerosene are measured and compared with section 2.7.

## Results and discussion

### Process chemistry

Sodium hydroxide readily reacts with naphthenic acids to form sodium naphthenate and water according to the following reaction [2, 18]:

1 $$ \mathrm{RCOOH}+\mathrm{NaOH}\rightleftarrows \mathrm{RCOONa}+{\mathrm{H}}_2 $$

(RCOOH represents naphthenic acids which consist of one or more saturated cyclic rings, alkylated at various positions, and a straight-chain carboxylated alkyl group)

Sodium hydroxide also reacts with H_2_S (if any) contained in the kerosene fraction in accordance with the following Eq. (2):

2 $$ 2\ \mathrm{NaOH}+{\mathrm{H}}_2\mathrm{S}\to {\mathrm{Na}}_2\mathrm{S}+2{\mathrm{H}}_2\mathrm{O} $$

The reaction of sodium hydroxide with naphthenic acids is a reversible reaction. This means that the operating parameters should be adjusted to keep the reaction in the forward direction.

### Effect of sodium hydroxide (caustic) concentration on the treatment process

**Fig. 2:**
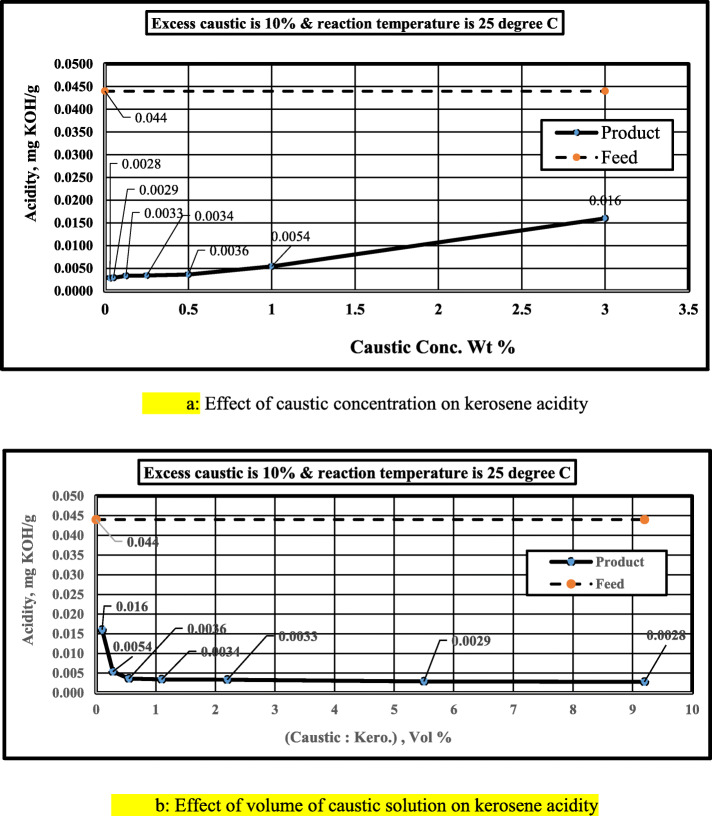
**a** Effect of caustic concentration on kerosene acidity. **b** Effect of volume of caustic solution on kerosene acidity

Figure 2a, b demonstrates the effect of caustic concentration on the acidity of treated kerosene. Diluted caustic solutions (with higher caustic volume) have more effect than the concentrated solutions (with less caustic volume). Table 4 indicates the volume of caustic solution associated with each concentration. The amount of NaOH molecules is the same in all solutions (27.5 mg), but concentration and volume are different.

As the caustic concentration increases, the stoichiometric caustic volume decreases. The reaction is more favorable with diluted solutions rather than concentrated ones. This behavior reflects that the reaction between sodium hydroxide and naphthenic acids is a diffusion-controlled chemical reaction.

The process of acids removal from kerosene in flow contactor or stirred tank mixer can be divided into two steps:

1. Diffusion of acids from kerosene (continuous phase) to the surface of droplets of the aqueous phase of sodium hydroxide (dispersed phase).
2. Reaction of acids with alkali in the droplets of the aqueous phase and removal of reaction products with the aqueous phase.

The diffusion step is controlled mainly by the surface area of droplets. The chemical reaction step in the droplets is mainly affected by the concentration of sodium hydroxide in the aqueous phase.

In industrial practice, a small volume of high concentration of NaOH aqueous solution is used (1–2 volumes of the aqueous solution to 100 volumes of kerosene). In this case, the surface area of droplets is very small, the diffusion rate is small (resistance is high), and the chemical reaction rate is high (resistance is small). Thus, the overall process is controlled by diffusion.

If the volume of the aqueous phase is increased by adding water only, the surface area of the dispersed phase increases, while the concentration of sodium hydroxide is decreased. This means the resistance of the diffusion step is decreased, while the resistance of reaction increases but the diffusion step is still controlling. This behavior continues with the dilution of NaOH solution, and the overall process of acid removal from kerosene is improved. At some point (optimum point of operation), the effect of the chemical reaction step becomes appreciable.

In our study, the point of maximum efficiency is at 5.5 volumes of the aqueous solution to 100 volumes of kerosene (using 110% of the theoretical amount of NaOH) and the efficiency is 91.8%. In actual refinery operations, using less volume of aqueous solution with a high concentration of NaOH (3 wt%), the efficiency is 63.6%.

For the given kerosene sample, the optimum caustic concentration is 0.5 wt% and using caustic solutions less than 0.5 wt% have a negligible effect on product acidity. From Table 4, the volume of caustic solution is 0.55% (by volume) of kerosene feed (with caustic concentration 0.5 wt%). In engineering applications, caustic solutions with 1–3 wt % are common according to kerosene feed acidity.

### Calculation of the amount of NaOH and cost impact of the diluted solutions

The conventional caustic wash process is an economically attractive process, since no catalyst or any special chemicals. From Table 4, all the prepared caustic solutions contain 27.5 mg of NaOH (including 10% excess).

Process efficiency with caustic solution of 3 wt% concentration

$$ =\left(0.044-0.016\right)/0.044=63.6\%. $$

Process efficiency with caustic solution of 0.5 wt% concentration

$$ =\left(0.044-0.0036\right)/0.044=91.8\%. $$

Required amount of NaOH to attain the same efficiency with caustic solution of 3 wt% concentration = 27.5 × 91.8/63.6 = 39.7 mg (including 10% excess).

Saving in caustic consumption with diluted caustic solutions of 0.5 wt% concentration

$$ =\left(1-\left(27.5/39.7\right)\right)\times 100=30.7\% $$

### Effect of excess caustic volume on the treatment process

**Fig. 3:**
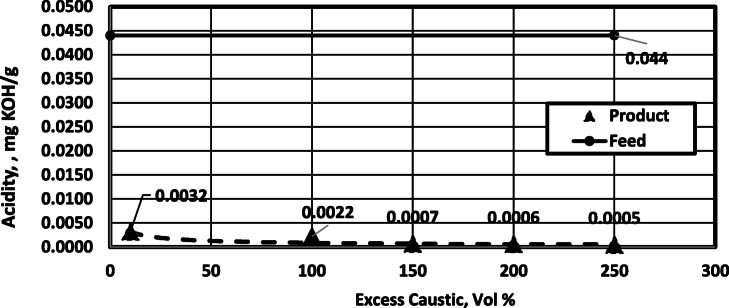
Effect of excess caustic solution on acidity

Figure 3 indicates the effect of excess caustic (at constant concentration 0.5 wt%) on product acidity. As shown, using more excess caustic solution has a slight effect on the acidity. For the given kerosene sample, 10% excess caustic is sufficient for the treatment. It is not economical to use a very large excess caustic solution with a minor effect on acidity. If we tried to use less than 10% excess caustic solution, higher product acidity would appear (lower process efficiency).

### Effect of the number of treatment stages on the treatment process

**Fig. 4:**
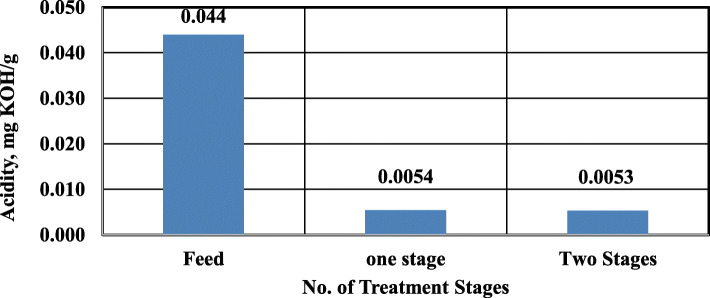
Effect of number of treatment stages on acidity

Figure 4 demonstrates the effect of a number of treatment stages on the acidity of treated kerosene. As shown, increasing the treatment stages has no effect. Therefore, one stage process is sufficient to remove acids.

### Effect of water wash on the treatment process

**Fig. 5:**
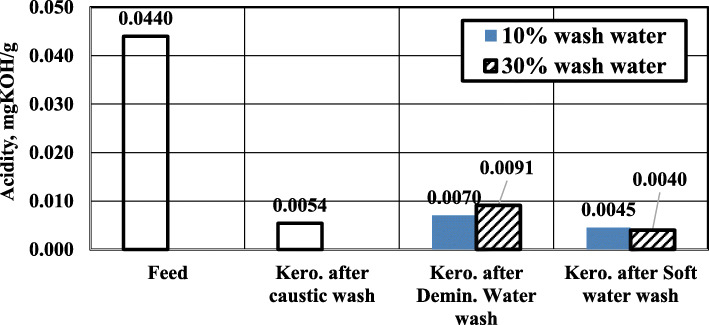
Effect of water wash on kerosene acidity

Figure 5 indicates the effect of water wash on the acidity of treated kerosene. As shown, washing caustic-treated kerosene with water has a slight effect on the acidity. Using demineralized water (with pH=7) has a slightly adverse effect on kerosene acidity. Increasing the demineralized water volume (with respect to kerosene feed volume) results in a slight increase in the acidity of the treated kerosene.

On the other hand, using alkaline soft water (with pH=9.44) has a slightly positive effect on kerosene acidity. Increasing the alkaline soft water volume results in a slight decrease in the acidity of the treated kerosene.

The abovementioned behavior can be interpreted by the effect of wash water pH. Demineralized water has pH =7 which is lower than the pH of soft water (soft water pH=9.44). As more demineralized water is added, some sodium naphthenates convert to naphthenic acid by the reverse reaction (Eq. 1).

On the other hand, alkaline soft water contains some alkalinity (carbonate alkalinity, Table 3) due to the addition of lime solution in the water treatment plant. Carbonates can react with existing acids in kerosene and reduce the kerosene acidity. Adding more volume of the alkaline soft water (with higher pH) increases the forward reaction of naphthenic acid to sodium naphthenate, which reduces the acidity.

**Fig. 6:**
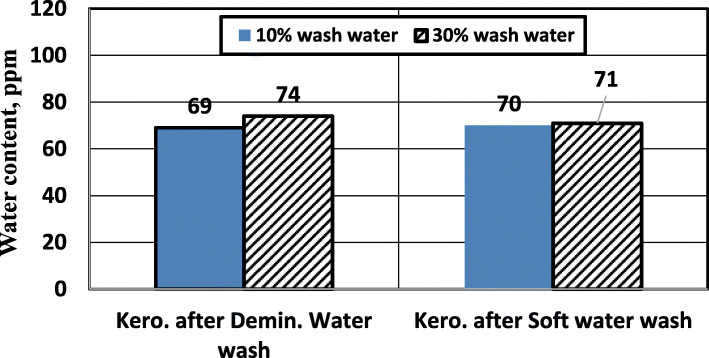
Effect of wash water (type and volume) on the water content of treated kerosene

Figure 6 shows the effect of water wash on the water content of treated kerosene. Increasing wash water volume has no noticeable effect on water content. Both types of wash water have the same effect on water content. For the given kerosene sample, washing the caustic-treated kerosene with alkaline soft water (10% of kerosene feed) is sufficient for the treatment.

## Conclusions

1. Two main steps are involved in the reaction between sodium hydroxide and naphthenic acids: Diffusion step of acids to the surface of droplets of sodium hydroxide; and reaction step of acids with alkali inside the droplets and removal of reaction products with the aqueous phase.
2. The results revealed that diluted caustic solutions are better than the concentrated ones. Thus, the reaction between sodium hydroxide and naphthenic acids is a diffusion-controlled chemical reaction; as the volume of the aqueous phase is increased by dilution, the surface area of the dispersed phase increased, and resistance of diffusion step is decreased, the overall rate of chemical reaction increased.
3. For the given kerosene sample, the optimum caustic concentration is 0.5 wt%. The volume of caustic solution is 0.55% (by volume) of kerosene feed.
4. For the given kerosene sample, saving in caustic consumption with diluted caustic solutions of 0.5 wt% concentration is 30.7% compared with caustic solutions of 3 wt%.
5. Using more excess caustic solution has a slight effect on kerosene acidity. For the given kerosene sample, 10% excess caustic (110% of the theoretical) is sufficient.
6. Performing the caustic treatment process in one stage is sufficient and the two-stage process has no effect on acidity.
7. Washing caustic-treated kerosene with demineralized water (pH=7) has a slight adverse effect on kerosene acidity. Increasing the demineralized water volume results in a slight increase in the acidity of the treated kerosene. Wash water should be slightly alkaline (pH 7.5–8) to prevent the reverse reaction of sodium naphthenates back into naphthenic acid.
8. Increasing wash water volume has no noticeable effect on the water content of treated kerosene. For the given kerosene sample, washing the caustic-treated kerosene with alkaline soft water (10% of kerosene feed) is sufficient for the treatment. Both types of wash water have the same effect on water content.

## Data Availability

All data presented and analyzed during the current study are reproducible with the provided information.

## Abbreviation

ASTM: American Society for Testing and Materials
